# Zika virus infection in Nicaraguan households

**DOI:** 10.1371/journal.pntd.0006518

**Published:** 2018-05-31

**Authors:** Raquel Burger-Calderon, Karla Gonzalez, Sergio Ojeda, José Victor Zambrana, Nery Sanchez, Cristhiam Cerpas Cruz, Harold Suazo Laguna, Fausto Bustos, Miguel Plazaola, Brenda Lopez Mercado, Douglas Elizondo, Sonia Arguello, Jairo Carey Monterrey, Andrea Nuñez, Josefina Coloma, Jesse J. Waggoner, Aubree Gordon, Guillermina Kuan, Angel Balmaseda, Eva Harris

**Affiliations:** 1 Division of Infectious Diseases and Vaccinology, School of Public Health, University of California, Berkeley, Berkeley, California, United States of America; 2 Sustainable Sciences Institute, Managua, Nicaragua; 3 Laboratorio Nacional de Virología, Centro Nacional de Diagnóstico y Referencia, Ministry of Health, Managua, Nicaragua; 4 Department of Medicine, Division of Infectious Diseases, School of Medicine, Emory University, Atlanta, Georgia, United States of America; 5 Department of Epidemiology, School of Public Health, University of Michigan, Michigan, United States of America; 6 Centro de Salud Sócrates Flores Vivas, Ministry of Health, Managua, Nicaragua; University of California, Los Angeles, UNITED STATES

## Abstract

Zika virus (ZIKV) infection recently caused major epidemics in the Americas and is linked to congenital birth defects and Guillain-Barré Syndrome. A pilot study of ZIKV infection in Nicaraguan households was conducted from August 31 to October 21, 2016, in Managua, Nicaragua. We enrolled 33 laboratory-confirmed Zika index cases and their household members (109 contacts) and followed them on days 3–4, 6–7, 9–10, and 21, collecting serum/plasma, urine, and saliva specimens along with clinical, demographic, and socio-economic status information. Collected samples were processed by rRT-PCR to determine viral load (VL) and duration of detectable ZIKV RNA in human bodily fluids. At enrollment, 11 (10%) contacts were ZIKV rRT-PCR-positive and 23 (21%) were positive by IgM antibodies; 3 incident cases were detected during the study period. Twenty of 33 (61%) index households had contacts with ZIKV infection, with an average of 1.9 (range 1–6) positive contacts per household, and in 60% of these households, ≥50% of the members were positive for ZIKV infection. Analysis of clinical information allowed us to estimate the symptomatic to asymptomatic (S:A) ratio of 14:23 (1:1.6) among the contacts, finding 62% of the infections to be asymptomatic. The maximum number of days during which ZIKV RNA was detected was 7 days post-symptom onset in saliva and serum/plasma and 22 days in urine. Overall, VL levels in serum/plasma, saliva, and urine specimens were comparable, with means of 5.6, 5.3 and 4.5 log_10_ copies/ml respectively, with serum attaining the highest VL peak at 8.1 log_10_ copies/ml. Detecting ZIKV RNA in saliva over a similar time-period and level as in serum/plasma indicates that saliva could potentially serve as a more accessible diagnostic sample. Finding the majority of infections to be asymptomatic emphasizes the importance of silent ZIKV transmission and helps inform public health interventions in the region and globally.

## Author summary

Zika virus (ZIKV) infection has become a major concern due to its association with congenital birth defects and Guillain-Barré Syndrome. We enrolled 33 laboratory-confirmed Zika cases (index cases) and their household members (109 contacts) in Managua, Nicaragua, and followed them for three weeks, collecting serum/plasma, urine and saliva specimens along with clinical, demographic, and socio-economic status information. We found that 61% of the index households had contacts with ZIKV infection, with an average of 1.9 (range 1–6) positive contacts per household, and in 60% of these households, ≥50% of the members were ZIKV-positive. Analysis of clinical information allowed estimating the symptomatic to asymptomatic (S:A) ratio of 14:23 (1:1.6) among the contacts. Finding 62% of the infections to be asymptomatic emphasizes the importance of silent transmission. Evaluating the maximum number of days during which ZIKV RNA was detectable showed that ZIKV was found up to 7 days post-symptom onset in serum/plasma and saliva and 22 days post-symptom onset in urine. Finding ZIKV RNA in saliva over a similar time period and concentration as serum/plasma indicates that saliva could potentially serve as a more accessible diagnostic sample. Overall, these data increase our understanding of ZIKV transmission and help inform public health interventions in the region and globally.

## Introduction

Zika virus (ZIKV) emergence in the Americas was first documented in northeastern Brazil in March of 2015 [1, 2], although the virus may already have been introduced in 2014 [3]. ZIKV spread rapidly throughout the Americas [4], and the World Health Organization (WHO) declared Zika a Public Health Emergency of International Concern in February of 2016 [4]. ZIKV infection in adults has been associated with Guillain-Barré Syndrome [4–6], and infection during pregnancy can have devastating effects, including severe congenital birth defects such as microcephaly and other neurological and developmental sequelae [7–10]. In Nicaragua, Zika was first confirmed in January of 2016, and the country experienced an explosive epidemic between June and September of 2016 [11]. As of August 2017, 48 countries and/or territories in the Americas reported autochthonous vector-borne ZIKV transmission [12].

Mathematical models suggest that a ZIKV-positive individual can lead to approximately 2–5 additional infections via mosquito transmission [13, 14]. ZIKV transmission is predominantly mosquito-borne, but sexual transmission has been reported as well [13, 15, 16]. Salivary transmission has been deemed possible but unlikely [13, 15–17]. Viral transmission is thought to depend on viral load (VL) in bodily fluids [18–20]; therefore, characterization of ZIKV VL kinetics in different compartments is of great interest. Although ZIKV viremia in humans is relatively low, ZIKV RNA has been detected in blood, urine, saliva, semen, and vaginal secretions [15, 21–23]. While reported duration of VL in bodily fluids varies greatly, ZIKV RNA measured by real-time reverse transcription-PCR (rRT-PCR) has revealed detectable viral RNA up to 76 days post-symptom onset in semen, 29 days in saliva and urine, and 14 days in vaginal secretions and serum [2, 21, 22, 24–32]. With the exception of one publication reporting detectable viral RNA in whole blood up to day 81 post-symptom onset [21], ZIKV was shed the longest in semen [22]. Symptomatic ZIKV infection has been associated with fever, conjunctivitis, rash, myalgia, arthralgia/arthritis, headache and fatigue, though fever may not always be present [32]. Estimates of asymptomatic ZIKV infections range from 27% to 82% [33–38], though more studies are needed.

Household-based index cluster study designs, where household or neighborhood contacts of viremic index cases are recruited [39], allow capture of symptomatic cases immediately at and before symptom onset, as well as capture of asymptomatic infections, household transmission events, geospatial spread of infection, and individual and household risk factors for infection, including entomological risk factors [40–43]. Additional data on household transmission, the percentage of asymptomatic ZIKV infections, and risk factors for ZIKV infection are essential to inform officials making critical public health decisions targeting prevention of ZIKV transmission; furthermore, little information has been published from Central America. The present index household study was implemented in Managua, Nicaragua, from August 31 to October 21 of 2016 and aimed to investigate household ZIKV transmission, maximum duration of viral RNA detection in bodily fluids, kinetics of ZIKV VL levels in bodily fluids, the symptomatic to asymptomatic (S:A) ratio, and risk factors for ZIKV infection.

## Methods

### Ethics statement

The Institutional Review Boards (IRB) of the University of California, Berkeley, and the Nicaraguan Ministry of Health approved the index cluster study protocol and the ongoing Nicaraguan Pediatric Dengue Cohort Study (PDCS). All adult participants provided written informed consent, and parents or legal guardians provided written informed consent for children participants. In addition, children between 6 and 18 years of age provided verbal assent.

### Study design

During the study period, rRT-PCR-confirmed Zika cases (potential index cases) from either the Nicaraguan PDCS [44] or the general population (National Surveillance System) who resided in the Health Center Sócrates Flores Vivas (HCSFV) catchment area were visited at their homes. The HCSFV catchment area consists of 17 neighborhoods within District II of Managua, the capital city of Nicaragua. Index cases (n = 33), along with all household members willing to participate in the study (contacts, n = 109), were enrolled into this study during the first household visit (day 1). Questionnaires were administered at the first visit and at subsequent visits on days 3–4 (visit 2), 6–7 (visit 3), 9–10 (visit 4), and 21 (visit 5), regardless of ZIKV infection status. The household questionnaire, addressing home-specific characteristics, was administered to the head of the household at the first home visit, and the individual questionnaire, addressing demographic, clinical history and socioeconomic status (SES)-associated factors, was administered to each household member. At each household visit, occurrence of symptoms was investigated by asking each study participant the question “Have you presented with any of the following symptoms?” followed by a list of common Zika symptoms. If a participant reported symptoms, the date of symptom onset and type of symptom(s) were recorded. During the follow-up visits (visits 2–5), the participants were asked to report symptoms since the last visit. Hence, the symptom questionnaire should cover prior occurrence of symptoms at the first visit and symptoms since the previous visit during subsequent follow-up visits. The participants were also asked “Have you been diagnosed with Zika in the past?”, assessing previous symptomatic infections. Blood (serum or plasma), urine, and saliva samples were collected at the visits as detailed in Table 1. For the index cases that were PDCS participants (85%, n = 28), the blood collection on the second and the final study visit corresponded to a programmed PDCS visit, and plasma was collected at these two time-points (see Table 1). For saliva collection, passive drool (minimum 2 ml) was collected into vials. Laboratory testing was performed at the National Virology Laboratory (NVL) of the Ministry of Health in Managua. All household visits were performed between August 31 and October 21 of 2016, which corresponded to the tail end of the national Zika epidemic in 2016.

**Table 1:** Sample collection schedule for index cases and contacts (n = 142) among enrolled households in Managua, Nicaragua, August to October, 2016.

	Visit 0^1^	Visit 1^2^ Day 1	Visit 2 Days 3/4	Visit 3 Days 6/7	Visit 4 Days 9/10	Visit 5 Day 21
**Index cases (n = 33)**						
Blood	X	X^3^				X^3^
Urine	X	X	X	X	X	X
Saliva	X	X	X	X	X	X
**Contacts (n = 109)**						
Blood		X	X			X
Urine		X	X	X	X	X
Saliva		X	X	X	X	X

^1^Applies only to index cases: 28 from PDCS and 5 from national surveillance (n = 33). No urine or saliva samples were available for surveillance cases.

^2^Enrollment day for index cases and contacts.

^3^For the 28 index cases that were PDCS participants [32], the second and final visits correspond to a plasma sample, whereas all the other collected blood samples are serum. The 5 surveillance index cases only have an enrollment (visit 1) and final (visit 5) serum sample available.

### Definitions

A suspected Zika case, whether captured from the PDCS or from national surveillance, was defined as a patient presenting with rash and one or more of the following symptoms: conjunctivitis, arthralgia, myalgia, and/or peri-articular edema, regardless of fever. A suspected Zika case became a potential index case once a ZIKV infection was confirmed by rRT-PCR in serum, urine and/or saliva.

Among contacts, ZIKV infection was defined by rRT-PCR and/or IgM positivity. IgM positivity was based on the initial and final sample and could be negative-positive (NP), positive-positive (PP) or positive-negative (PN). A symptomatic ZIKV-positive contact had at least one of the following symptoms: rash, fever, conjunctivitis, arthralgia, myalgia, peri-articular edema and/or headache.

Days post-symptom onset were calculated as following: (sample collection date)–(symptom onset date) + 1. Hence, day 1 post-symptom onset is equivalent to the day the symptoms initiated.

### Nicaraguan Pediatric Dengue Cohort Study (PDCS)

The ongoing PDCS, which served as the parent study, was established in Managua in 2004 and follows ~3,700 2-to-14 year-old children who reside in the HCSFV catchment area. The HCSFV provides health care to all study participants. Acute-phase (days 1–5 since symptom onset) and convalescent-phase (days 14–21) blood, saliva, and urine samples are collected from suspected dengue, chikungunya, and Zika cases, along with clinical information. Suspected dengue or chikungunya cases are patients with fever (or feverish) and two or more of the following symptoms: headache, retroocular pain, myalgia, arthralgia, rash, positive tourniquet test and/or leukopenia. Acute-phase serum samples are tested for ZIKV, chikungunya virus (CHIKV) and dengue virus (DENV) simultaneously using a multiplex rRT-PCR assay [45]. Paired acute- and convalescent-phase blood samples are evaluated for dengue, chikungunya, and Zika IgM antibodies as well as total (primarily IgG) antibodies [44, 46–48]

### Laboratory assays

All diagnostic assays were performed at the NVL. Biological specimens of index cases and contacts were transported from the HCSFV to the NVL the same day as collection or, if the patient presented to the HCSFV after normal business hours, the following day. Samples were kept at 4°C until transported to the NVL for processing and storage at -80°C.

### Multiplex rRT-PCR

RNA was extracted from 140 ul of blood, 200 ul of urine, or 200 ul of saliva specimens using the QIAamp Viral RNA Mini Kit (Qiagen, Valencia, CA) and amplified using the ZCD rRT-PCR assay targeting ZIKV, DENV and CHIKV [45]. Testing was performed on an ABI 7500 Fast instrument (Applied Biosystems, Foster City, CA) in 25-ul reactions of the SuperScript III Platinum One-Step qRT-PCR kit (Life Technologies, Carlsbad, CA) with 5 ul of RNA template, performed as previously described [45]. Each plate was processed with negative and positive controls for ZIKV, CHIKV, and DENV. Standard curves were prepared using quantitated ssDNA (Integrated DNA Technologies) containing the target sequences for ZIKV amplification. The concentration of RNA in the eluate (expressed as log_10_ copies/ul) was calculated from the linear regression equation for the standard curve. Viremia in log_10_ copies/ml was then calculated while accounting for the volume used for extraction. For ZIKV quantitation, a 4-point standard curve (8.0, 6.0, 4.0, and 2.0 log_10_ copies/μL of eluate) was included on every plate in duplicate. Urine samples from 9 contacts were extracted at higher volumes (1 ml instead of 200 ul) to concentrate the viral RNA. Volume variation was accounted for in the viral load (VL) calculations (S1 Table).

### IgM-capture enzyme-linked immunosorbent assay (ELISA)

An in-house single-dilution IgM-capture enzyme-linked immunosorbent assay (ELISA) [48] was performed to detect ZIKV-specific IgM antibodies on paired day 1 and day 21 serum/plasma samples. This method is similar to that described for detection of DENV-specific IgM [49] or CHIKV-specific IgM [50] and was adapted for detection of ZIKV IgM antibodies using a ZIKV envelope (E) domain III-specific monoclonal antibody (mAb), ZKA64, kindly donated by D. Corti (Humabs Biomed SA) [51]. Briefly, wells were coated with goat anti-human IgM antibody overnight and then blocked using 4% bovine serum albumin (BSA) diluted in 0.05% PBS-T (1X phosphate-buffered saline containing 0.05% Tween 20). Subsequently, antigen produced from ZIKV-infected suckling mouse brain and extracted via the sucrose-acetone method [52] was added. Next, a single dilution of samples and controls (1:20) was added in duplicate, followed by anti-ZIKV mAb ZKA64 conjugated to horseradish peroxidase. After every incubation step, the wells were washed 4–5 times with 0.05% PBS-T. In the final step, 3’3’-5’5’-tetramethylbenzidine (TMB) was used to produce a colorimetric reaction that was terminated with 12.5% sulfuric acid. The cut-off was determined as 6.6 times the average of negative controls. All of the samples with absorbance values above the cut-off were considered positive. Evaluation with an extensive panel of Zika, dengue, and negative samples yielded a sensitivity of 94.5% and specificity of 85.6% [48].

### Entomological visits and analysis

Households were surveyed on visit 4 to assess entomological indicators. Water containers and potential breeding sites were inspected, and larvae and pupae were collected, speciated, and enumerated by experts in collaboration with the Department of Entomology at the NVL. Adult mosquitoes were captured in the interior and exterior of the houses using Prokopack backpack aspirators [53, 54]. Captured live adults were enumerated and grouped by sex and species (*Aedes aegypti*, *Aedes albopictus* and *Culex quinquefasciatus)* for each household. Female bodies were homogenized after removal of legs and wings in 600 ul RNAlater^®^ solution (Sigma Aldrich) to stabilize RNA for rRT-PCR analysis, and the homogenate was frozen at -80°C. RNA was extracted from 200 ul of the homogenate using the QIAamp Viral RNA Mini Kit (Qiagen) and analyzed for ZIKV, DENV and CHIKV positivity by rRT-PCR (see above).

### Statistical analysis

ZIKV positivity and mean VL levels, along with standard deviations (SD; error bars), were calculated and graphed in R Statistical Software V.3.4.2 [55]. Duration of RNA detection was determined based on the maximum number of days duration of ZIKV RNA detection for each bodily fluid, where the days were based on symptom onset (see calculation of days post-symptom onset under the “Definitions” section above). The sample size for each time-point (listed by bodily fluid in table below graph) was based on days post-symptom onset. Several time-points had few samples due to variation of days post-symptom onset at which the participants were enrolled. The first time-point of 5 surveillance index cases and 4 ZIKV rRT-PCR-positive contacts with no symptoms were excluded from the ZIKV positivity and VL analysis due to missing VL data and day of symptom onset information, respectively. Further, an extreme outlier for urine VL was excluded (8 days post-symptom onset, 8.9 log_10_ copies/ml). Percent positivity was defined as the percent of positive samples at specified days post-symptom onset for each bodily fluid.

The symptomatic infection rate attributable to ZIKV infection for the contacts (n = 109) was calculated by subtracting the percent of ZIKV-negative contacts with ZIKV-associated symptoms (12/72 = 17%) from the percent of ZIKV-positive contacts with ZIKV-associated symptoms (14/37 = 38%) [43]. Risk ratios (RR) were calculated using modified Poisson regression [56] in the context of a general estimating equations (GEE) model to identify associations between risk factors and contacts being positive for ZIKV infection by either rRT-PCR and/or IgM ELISA. The GEE model accounts for clustering due to the household-based study design. An exchangeable correlation structure was assumed, and confidence intervals were calculated using a robust (sandwich) variance estimator. The following were considered risk factors of interest for ZIKV infection: sex, age, household size, water faucet location outside of the house, on-site water storage, and recognizing mosquito larvae or pupae (all binary). Lack of variation in the study population precluded meaningful analysis of fumigation, abatement use, trash collection services, water services and repellent use reports. Potential confounders were assessed based on a literature-informed directed acyclic graph (DAG; S1 Fig). Due to the small sample size and minimal variation of the confounders among participants, the multivariate analysis was adjusted for a single SES proxy indicator; namely owning a refrigerator. All analyses were conducted using SAS version 9.4 (SAS Institute, Cary NC). The map was constructed in ArcGIS ArcMap V.10.3 (Environmental Systems Research Institute, Redlands CA).

## Results

### Identification and spatial distribution of index cases and contacts

**Fig 1:**
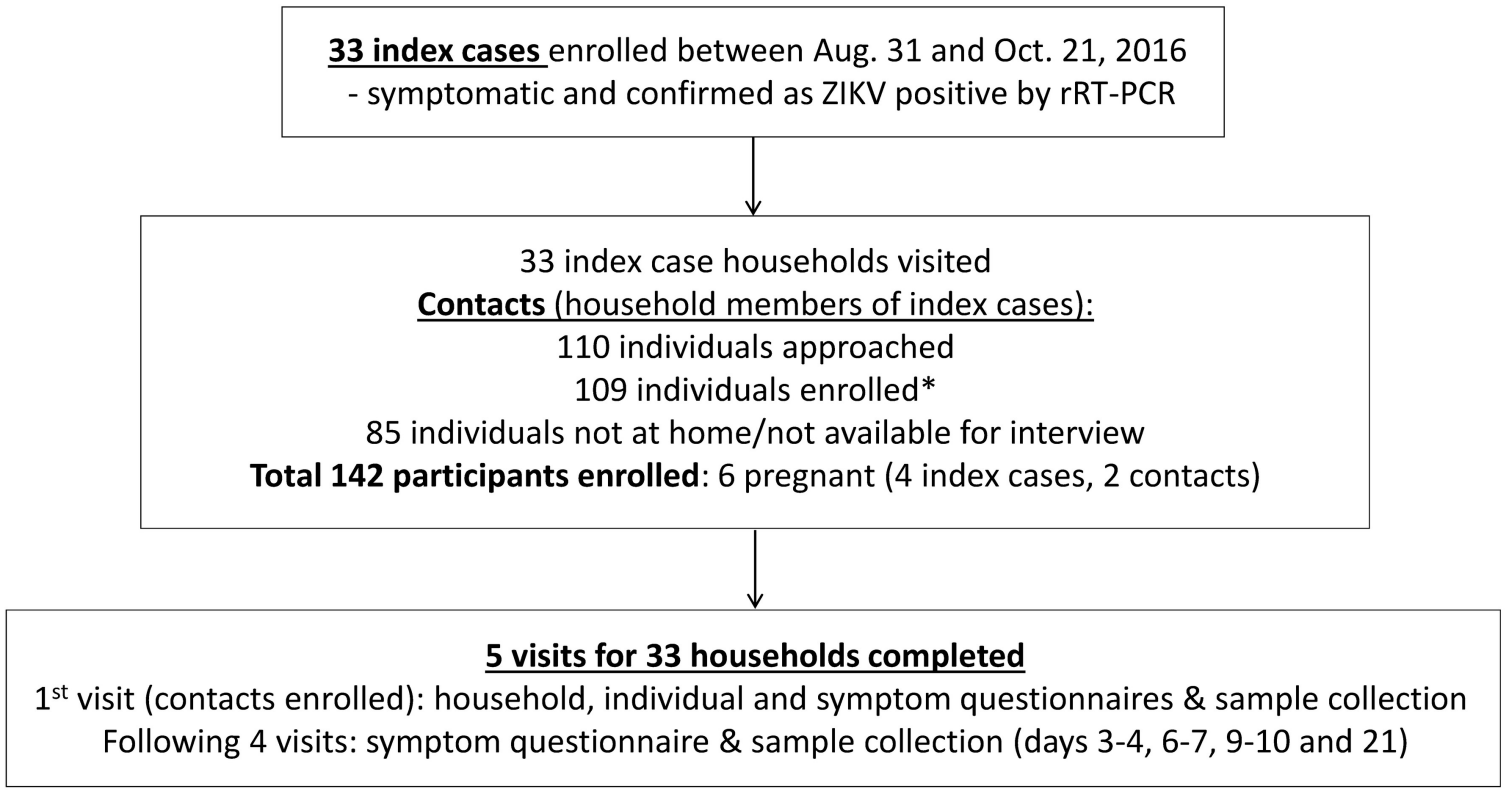
Flow chart of study design and enrollment for index cluster study of ZIKV infection in Managua, Nicaragua, 2016. *One enrolled contact did not answer the individual questionnaire but fulfilled the other study requirements such as symptom questionnaire and sample donation.

**Fig 2:**
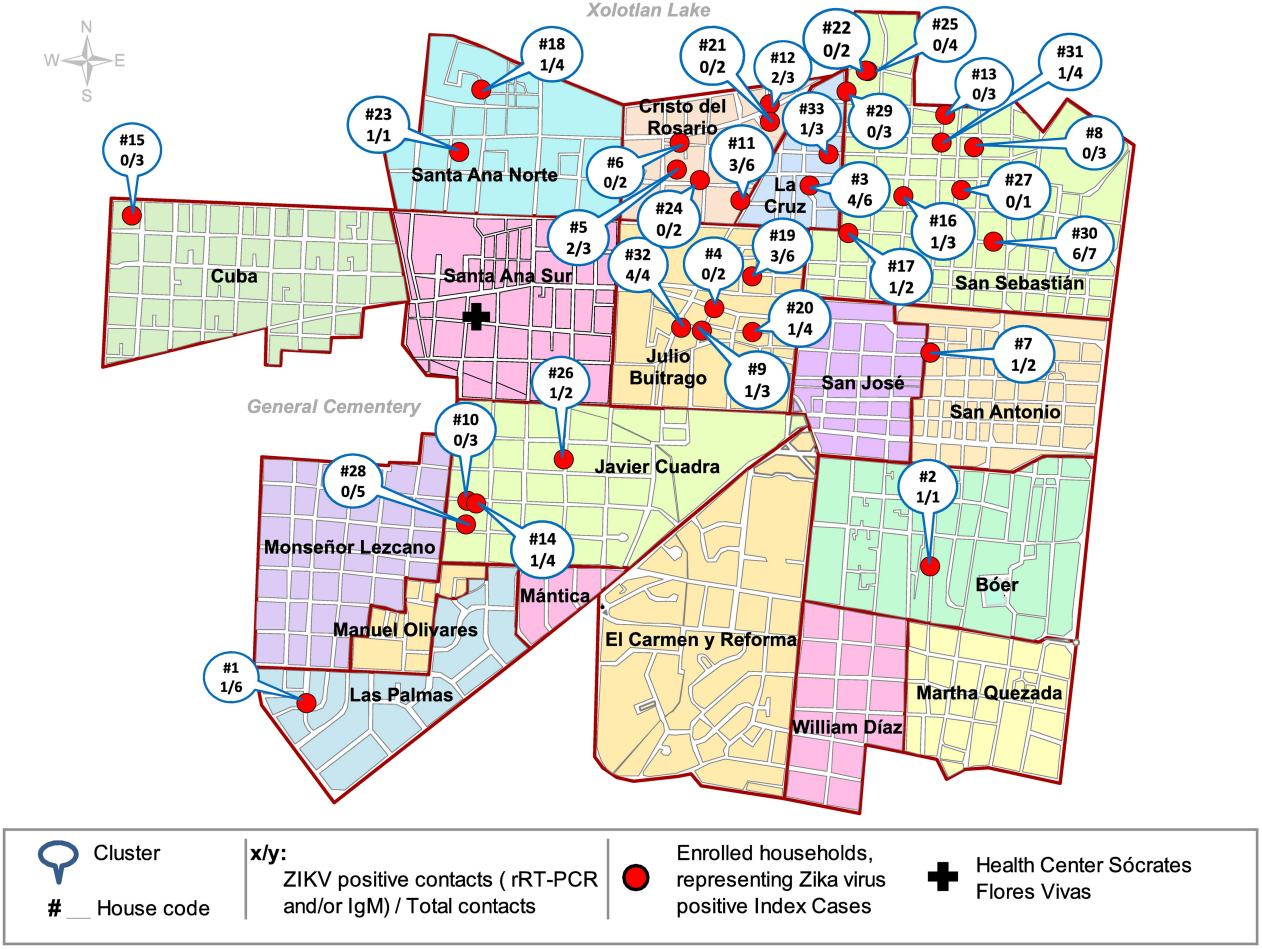
Geographic distribution of enrolled households, based on ZIKV-positive index cases from the Health Center Sócrates Flores Vivas, Managua, Nicaragua, August to October, 2016.

A total of 33 symptomatic rRT-PCR ZIKV-positive index cases were enrolled between August 31 and October 21 of 2016 (Fig 1). Each index case represented one household located within the HCSFV catchment area, which includes 17 neighborhoods in District II of Managua (Fig 2). The majority of the index cases (85%, n = 28) were participants of the Nicaraguan PDCS [44]. The remaining 5 index cases were captured as part of the national surveillance system; of these, 4 were pregnant women. The Ministry of Health prioritized Zika diagnosis for pregnant women upon symptom onset due to the known congenital risk factors. A total of 109 contacts, including two pregnant women, were enrolled at the first household visit from the 33 households, corresponding to a total of 142 participants. While all subjects approached agreed to participate in the study and there was no loss to follow-up, a total of 85 household members (37%) who were reported to be living in the respective home by the head of the household, but were not at home during the enrollment visit, were not interviewed or enrolled (S2 Table). One enrolled contact did not answer the individual questionnaire but fulfilled the other study requirements, including the symptom questionnaire and sample donation. The number of enrolled contacts per household ranged from 1 to 7, with an average of 3.3 ± SD of 1.6. The average age of index cases and contacts was 11.4 (±7.8, range 2–37) and 30.7 (±20.6, range 1–79) years, respectively (Table 2). The index cases tended to be younger since the majority were PDCS participants. Both index cases and contacts were 70% female. The heads of the 33 enrolled households reported little variation with respect to SES-associated household characteristics: 100% reported zinc as the roof material; 100% reported to be the house owners; 97% (n = 32) reported the house floor material to be concrete, brick, and/or ceramic tiles, and only one reported a dirt floor; none reported owning an air conditioner; and 67% (n = 22) reported owning a refrigerator.

**Table 2:** Demographic characteristics, ZIKV infection and symptomatic Zika status among study participants in Managua, Nicaragua, August to October, 2016.

Characteristics	Index cases (n = 33) n (%)	Contacts (n = 109) n (%)	At enrollment ZIKV IgM+ (n = 23) n (%)^1^	At enrollment ZIKV rRT-PCR+ (n = 11) n (%)^2^	Incident cases (n = 3) n (%)^3^
Age (years)					
< 15	28 (85)	29 (27)	6 (26)	6 (55)	1 (33)
≥ 15	5 (15)	80 (73)	17 (74)	5 (45)	2 (67)
Mean	11.4	30.7			
Gender					
Male	10 (30)	33 (30)	7 (30)	3 (27)	2 (67)
Female	23 (70)	76 (70)	16 (70)	8 (73)	1 (33)
Symptoms in ZIKV-positive contacts^4^					
Yes			6 (26)	7 (64)	1 (33)
No			17 (74)	4 (36)	2 (67)

^1^ Zika IgM-positive contacts at enrollment = single Zika IgM-positive result at visit 1.

^2^ZIKV rRT-PCR-positive contacts at enrollment (7 by PCR only and 4 by both rRT-PCR and IgM).

^3^Three post-enrollment ZIKV infections, hence incident cases, among contacts were captured by IgM conversion, but all collected bodily fluid samples remained rRT-PCR-negative throughout.

^4^Symptoms for ZIKV-positive contacts (n = 37) by assay (IgM or rRT-PCR) and time-point (at or post-enrollment). All enrolled ZIKV rRT-PCR confirmed index cases (n = 33) were symptomatic.

### ZIKV infection and transmission

Serum/plasma, urine, and saliva samples were collected from index cases and contacts during a total of 165 household visits (5 visits to 33 households, see Table 1) and analyzed by molecular and serological assays as described in the Methods section. At enrollment, 34 contacts (31%) were positive for ZIKV infection, of which 11 (32%) were acutely infected (Table 2; 7 rRT-PCR-positive only and 4 rRT-PCR- and IgM-positive). The remaining 23 (68%) were positive by IgM only, indicating a recent ZIKV infection. The majority of the cases that were ZIKV-positive at enrollment were 15 years or older (n = 22, 65%) and female (n = 24, 71%). Three ZIKV incident infections were captured by IgM seroconversion among the contacts who were initially Zika-naïve, two of whom were 15 years or older and one female. The three seroconversions were located in different households. Overall, a total of 37 (34%) positive contacts were identified during the study period, 34 at enrollment and 3 post-enrollment, corresponding to 20 households. Hence, 20 households (61%) recorded ZIKV-infected contacts, with an average of 1.9 positives per household (±1.4, range: 1–6) among enrolled household members (Table 3, S2 Table). The corresponding ZIKV positivity per household ranged from 17% to 100%, with an average of 53% (±26.4), and in 60% of these households, ≥50% of the members tested were ZIKV-positive (Table 3, S2 Table). CHIKV and DENV rRT-PCR was performed on each sample analyzed, as part of the rRT-PCR multiplex assay, but no positive results were obtained.

**Table 3:** ZIKV infection positivity and percent symptomatic contacts among enrolled households in Managua, Nicaragua, August to October, 2016.

#	House	Pos. Contacts^1^	Total Contacts	% Positivity	Sympt. Pos. Contacts^2^	% Sympto-matic^3^	Incident cases
1	1	1	6	17	1	100	
2	14	1	4	25	1	100	
3	18	1	4	25	1	100	
4	20	1	4	25	0	0	
5	31	1	4	25	0	0	
6	9	1	3	33	1	100	X
7	16	1	3	33	0	0	
8	33	1	3	33	0	0	
9	7	1	2	50	0	0	
10	17	1	2	50	0	0	X
11	26	1	2	50	0	0	
12	11	3	6	50	3	100	
13	19	3	6	50	1	33	
14	5	2	3	67	1	50	
15	12	2	3	67	1	50	
16	3	4	6	67	1	25	
17	30	6	7	86	2	33	X
18	2	1	1	100	0	0	
19	23	1	1	100	1	100	
20	32	4	4	100	0	0	

^1^ ZIKV-positive contacts: laboratory-confirmed by ZIKV RT-PCR and/or IgM ELISA.

^2^Symptomatic ZIKV-positive contacts: laboratory-confirmed ZIKV RT-PCR- and/or IgM-positive contacts that reported ZIKV-associated symptoms.

^3^ Percent of symptomatic cases among ZIKV-positive contacts in each household.

### Detectable ZIKV RNA in bodily fluids

**Fig 3:**
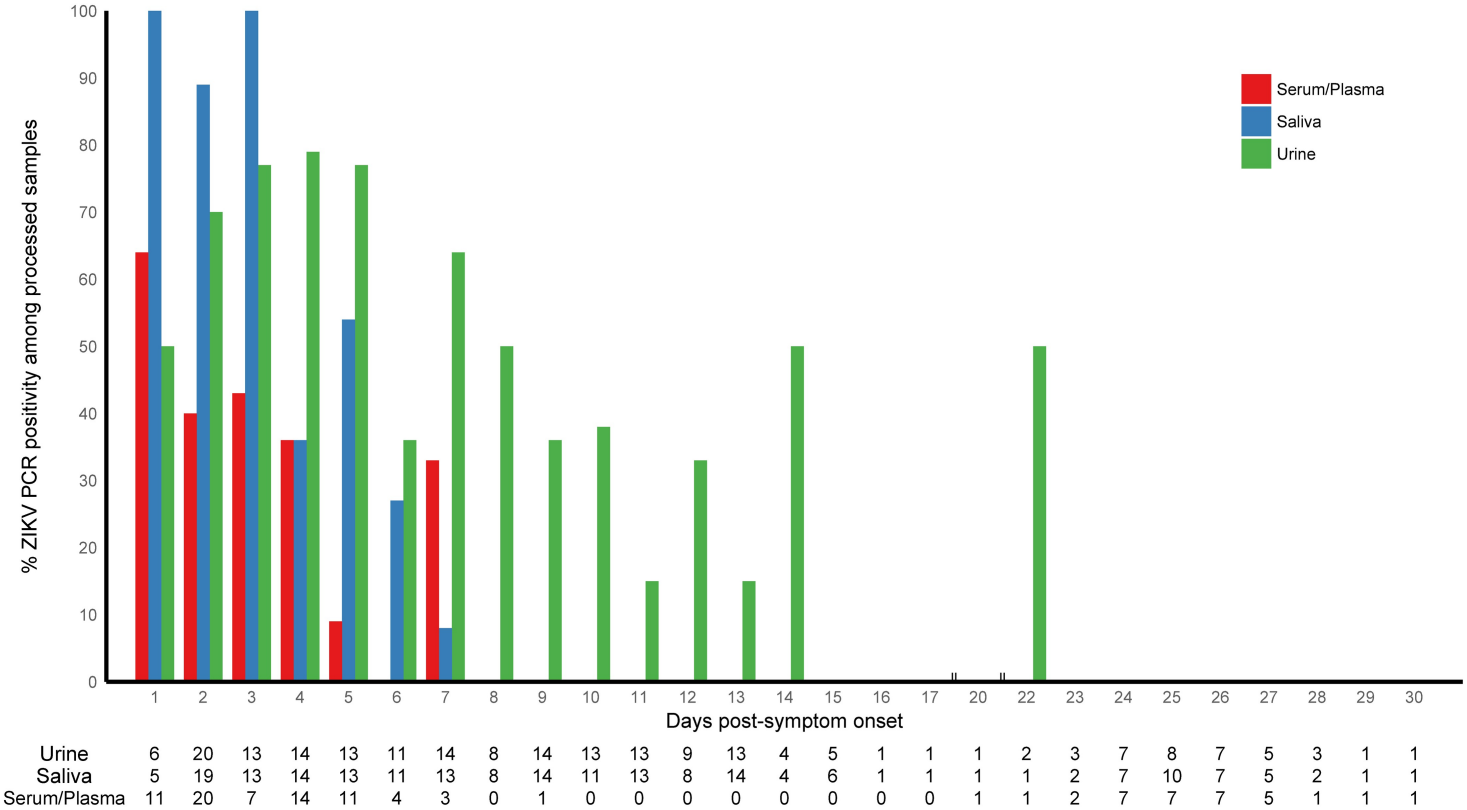
ZIKV rRT-PCR positivity in bodily fluid samples by day post-symptom onset, collected in enrolled households in Managua, August to October, 2016. The graph depicts ZIKV rRT-PCR positivity (%) in serum/plasma, saliva and urine over days post-symptom onset. The numbers presented in the table at the bottom are the total number of specimens processed for each bodily fluid at each time-point.

Despite the modest number of rRT-PCR confirmed ZIKV infections captured (33 index cases and 11 contacts), a total of 522 bodily fluid samples were processed by rRT-PCR (105 serum/plasma, 206 saliva, and 212 urine, Fig 3 and S1 and S3 Tables). All 44 rRT-PCR-confirmed ZIKV infections were positive at the entry visit: 13 (29%) were positive for ZIKV RNA in urine and saliva; 11 (25%) were positive in serum and saliva; 10 (23%) in serum only; 5 (11%) in urine only; 2 (5%) in serum, saliva and urine; 2 (5%) in saliva only; and 1 (2%) in serum and urine. Percent positivity (see Methods) declined over time for all fluids (Fig 3), with the exception of several time-points where the sample size was very small (numbers presented in the table at the bottom of Fig 3 are total number of specimens processed). Among rRT-PCR-positive subjects with Zika-associated symptoms, the maximum number of days duration of ZIKV RNA detection was 7 days post-symptom onset in serum/plasma and saliva and 22 days in urine (Fig 3). The mean number of days duration of ZIKV RNA detection was 2.56 (SD: 1.53), 3.10 (SD: 1.50) and 5.94 (SD: 3.80) days post-symptom onset in serum/plasma, saliva and urine, respectively (S3 Table).

**Fig 4:**
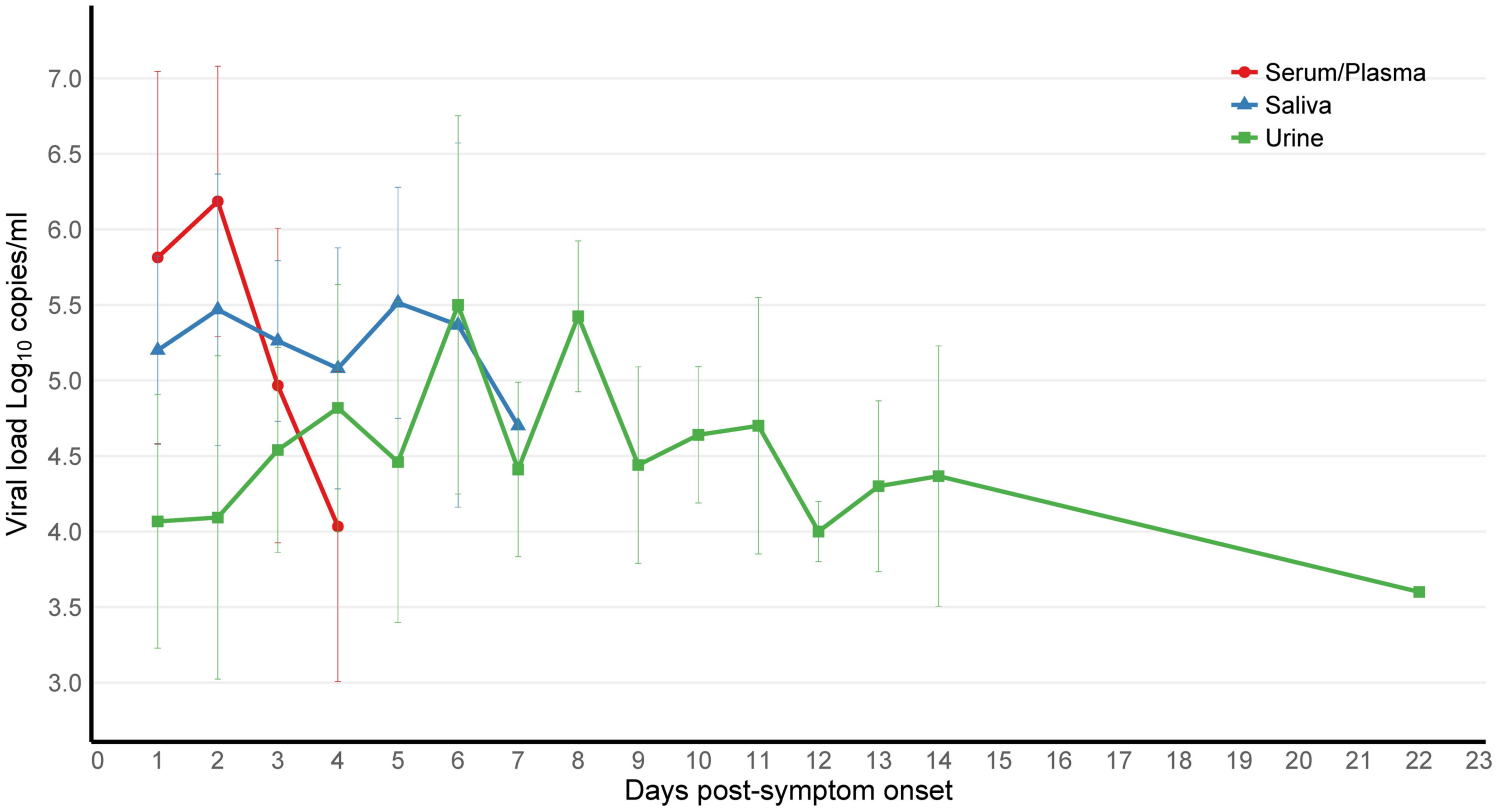
ZIKV viral load kinetics over time in bodily fluid samples (serum/plasma, saliva and urine) by day post-symptom onset, collected in enrolled households in Managua, August to October, 2016. Mean and standard deviation of viral load in bodily fluids plotted according to day post-symptom onset.

Overall, VL tended to decrease over time, and mean VL in the serum/plasma, saliva, and urine specimens was comparable, with 5.6 (± 1.2, range 2.9–8.1), 5.3 (± 0.8, range 3.2–6.9) and 4.5 (± 1.0, range 1.7–6.6) log_10_ copies/ml, respectively, with serum attaining the highest VL peak at 8.1 log_10_ copies/ml (Fig 4). Interestingly, one contact had a serum VL of 6.7 log_10_ copies/ml two days before symptom onset (data point excluded from Fig 4). Previous reports suggest prolonged maternal viremia in pregnant women [57–60]. While our sample size was very small, the four ZIKV rRT-PCR confirmed pregnant women in our study had a mean VL of 4.4 log_10_ copies/ml ZIKV in urine (up to 7 days post-symptom onset) and mean VL of 3.5 log_10_ copies/ml in serum/plasma (up to 5 days), comparable to non-pregnant subjects; however, the women were only followed for 21 days.

### Symptomatic to asymptomatic (S:A) ratio

On average, the symptomatic index and contact cases (n = 40) were enrolled 2.2 days post-symptom onset (±1.5, range -1 to 7). Among the 37 Zika-positive contacts, 14 were symptomatic (38%), while 12 of 72 ZIKV-negative contacts reported similar symptoms (17%), resulting in a 21% symptomatic infection rate attributable to ZIKV infection (see Methods). Among the 14 symptomatic Zika-positive contacts (7 rRT-PCR and 7 IgM-positive, see Table 2), the following Zika-associated symptoms were recorded: rash (57%), arthralgia (43%), fever (36%) and headache (29%). While conjunctivitis was listed on the symptom questionnaire, it was not reported by any of the Zika cases. These results are consistent with the parent PDCS cohort, where only 6% of Zika cases reported conjunctivitis during the epidemic. Several other symptoms, such as cough, shivers, difficulty breathing, ophthalmoplegia, nausea, and pruritus were reported (S4 Table). Furthermore, a symptomatic to asymptomatic (S:A) ratio of 14:23 (1:1.6) was determined among the contacts. Of the 23 asymptomatic ZIKV-positive contacts, 4 were rRT-PCR- and IgM-positive, and 19 were positive by serology only (Table 2). Of the 20 households with ZIKV-positive contacts, 11 (55%) contained symptomatic contacts (Table 3). The percent of symptomatic cases among ZIKV-positive contacts in the households ranged from 25–100%, with an average of 40% (± 43.8).

### Entomological analysis

An entomological survey was completed for each household during visit 4. Twenty-three (70%) of the inspected households were positive for adult mosquitoes, and a total of 109 specimens were captured. Of these, 85 (78%) were identified as *Aedes (Ae*.*) aegypti* and 24 (22%) as *Culex quinquefasciatus*. All 41 of the captured female *Ae*. *aegypti* tested negative for ZIKV, CHIKV, and DENV RNA by rRT-PCR. Seven (21%) of the inspected households were positive for *Ae*. *aegypti* larvae and/or pupae (total of 615 identified specimens). The majority of households (52%, n = 17) had ZIKV-infected residents as well as *Ae*. *aegypti* mosquitoes and/or infested water containers on the premises, whereas only 3 households (9%) had Zika-positive residents and were found to be free of *Ae*. *aegypti* mosquitoes and pupae/larvae. The remaining households were either mosquito/larvae/pupae-positive and Zika-negative (24%, n = 8) or mosquito/larvae/pupae-negative and Zika-negative (15%, n = 5).

### Risk factor analysis for ZIKV infection

All households reported abatement of water containers with Temephos in and around the household. A total of 29 (88%) households reported Temephos being deposited four times per year (abatement), and all households reported fumigation. The Ministry of Health provided these vector control measures at no cost. All households reported regular trash collection service, and only 12% (n = 4) reported having problems with regular water supply, thus precluding meaningful analysis. Repellent use was rare, with only 8% (n = 9) reporting use; 5 female and 4 male. Sex, age (being ≥15 years old), household size (≥4 contacts in a household), having a water faucet located outside of the house, and recognizing mosquito larvae or pupae were not statistically associated with ZIKV infection (Table 4). The risk of being ZIKV-positive among those who reported storing water on site, adjusted for owning a refrigerator (SES indicator), was 2.32 (95%CI: 1.26, 4.28) times the risk of being ZIKV-positive among those who reported not storing water, across households.

**Table 4:** Risk factor analysis for ZIKV infection among contacts (n = 109).

Risk Factor (binary)	N (%)	RR (95% CI)^1^	aRR (95% CI)^2^
Female	76 (70)	0.79 (0.50, 1.24)	0.80 (0.51, 1.26)
Age, ≥15 yrs	80 (73)	0.70 (0.42, 1.16)	0.68 (0.41, 1.13)
Contacts ≥ 4^3^	60 (55)	1.61 (0.84, 3.09)	1.60 (0.83, 3.06)
Owning refrigerator	79 (73)	1.15 (0.59, 2.25)	NA^4^
On-site water storage	35 (32)	**2.27 (1.24, 4.16)**	**2.32 (1.26, 4.28)**
Outside faucet location	64 (59)	0.74 (0.38, 1.45)	0.74 (0.38, 1.47)
Recognizing larvae/pupae	67 (62)	0.85 (0.43, 1.66)	0.85 (0.43, 1.66)

Note: Bold indicates significance at p<0.05

^1^Risk Ratio and 95% Confidence Interval, across households.

^2^Risk Ratio adjusted for owning refrigerator (SES indicator) and 95% Confidence Interval, across households.

^3^Contacts living in a household with 4 or more contacts.

^4^Not applicable.

## Discussion

This investigation of ZIKV household transmission enabled determination of the S:A ratio, duration of detectable ZIKV RNA, kinetics of ZIKV VL in bodily fluids, and risk factors for ZIKV infection. The index cluster study design enables capture of asymptomatic infections, since symptom data are actively sought, in this case approximately every 3–4 days for the first 9–10 days and then again 11–12 days later. We calculated an S:A ratio of 1:1.6, corresponding to 62% asymptomatic ZIKV infections, which is consistent with published studies [33–38]. Previous reports found the percent of asymptomatic ZIKV infections to range from 27% (by systematically screening returning travelers from Suriname) [36] to 82% (based on a household serological survey during the Yap Island Zika outbreak, where 18% of Zika IgM-positive individuals reported symptoms likely attributable to ZIKV infection [34]). These findings, along with our detection of relatively high serum VL before symptom onset, imply that asymptomatic infection is an important factor in ZIKV transmission. Further, our study found that 61% of the households had contacts with ZIKV infection, with an average of 1.9 (range 1–6) positive contacts per household. In 60% of the households, ≥50% of the members were positive for ZIKV infection. Thus, the majority of the households and the majority of household members of the ZIKV-positive index cases were positive for ZIKV infection, suggesting that ZIKV transmission was widespread among the enrolled households in urban Managua, Nicaragua.

Analysis of the magnitude of ZIKV VL in different bodily fluids revealed overall similar VL values in serum/plasma, saliva, and urine specimens, with serum attaining the highest peak VL (8.1 log_10_ copies/ml), similar to previously reported ZIKV VL levels [37]. ZIKV RNA was detected in all three bodily fluids from the first day of symptom onset, while VL decreased over time. Interestingly, one contact had relatively high serum VL (6.7 log_10_ copies/ml) two days before symptom onset. This is consistent with the previously estimated incubation period of 3–14 days [61], and viremia prior to symptom onset has been reported in blood donors (1–6 days) in Martinique [37]. As for duration, our data revealed detectable viral RNA up to 7 days post-symptom onset in serum/plasma and saliva and up to 22 days in urine. Hence, ZIKV was present the longest in urine, consistent with other publications [25, 26, 29, 33]. While reported duration of ZIKV RNA detection in bodily fluids varies greatly, studies have found detection times up to 29 days post-symptom onset in saliva and urine and up to 14 days in serum [2, 21, 22, 24–32]. Given that all captured index cases were symptomatic and that 33 of the 44 rRT-PCR-positive cases were index cases, it is possible that infection rates and viral loads detected differ from studies conducted among asymptomatic cases, such as viremic blood donors.

In our analysis, all index cases that had initial saliva samples (n = 26) would have been captured as ZIKV rRT-PCR-positive with acute saliva samples only. Furthermore, saliva and serum/plasma had similar mean VL levels and mean days of ZIKV detection. Other studies detected ZIKV RNA more frequently in saliva than in serum samples, while the mean number of days duration from symptom onset was the same (3.5 days) [30, 32]. Another study found that while no cases were exclusively positive in saliva, when screening serum, whole blood, urine and saliva, testing saliva instead of urine or saliva did not lower the diagnostic sensitivity during acute infection [24]. Hence, saliva, an accessible and non-invasive sample, may be an underrated and feasible diagnostic specimen for ZIKV rRT-PCR testing in suspected symptomatic cases, although it does not extend the window of detection beyond that of serum or whole blood [33].

It is known that water storage containers, present mostly due to intermittent water supply, create mosquito breeding sites and thus increase risk of virus transmission [62]. Entomological visits conducted on Yap Island during the Zika outbreak in 2007 found that 87% of the surveyed households had larvae- or pupae-infested water containers, although no viral RNA was detected in mosquitoes and/or immature stages [34]. In our study, 21% of the inspected households had water containers infested with *Ae*. *aegypti* larvae or pupae. As for adult mosquitoes, 70% of the households were positive, but none of the adult female *Ae*. *aegypti* captured during the entomological visits were positive for ZIKV RNA. However, these results could have been affected by the intensive government vector control activities (fumigation and abatement) implemented concurrently with the study. Although analysis of Zika-positive households and entomological indicators revealed no significant associations, 52% of the households were positive for both ZIKV-infected residents and the presence of mosquitoes, larvae and/or pupae.

A major strength of the study was the number of visits per household– 5 visits in a time span of 21 days—that enabled close monitoring of the duration of ZIKV RNA detection and symptoms. The study also experienced several limitations. The Nicaraguan Zika epidemic lasted from June to October of 2016, with peak incidence in July and August. Hence, the present study was conducted during the tail end of the epidemic, which is likely the reason that few transmission events were captured. Because of the antigenic relatedness of flaviviruses, particularly DENV and ZIKV, the IgM ELISA can potentially display cross-reactivity between ZIKV- and DENV-induced antibodies. However, comprehensive evaluation of the in-house MAC-ELISA with an extensive panel of specimens yielded a sensitivity of 94.5% and specificity of 85.6% when compared to rRT-PCR-confirmed Zika or dengue cases and flavivirus-naïve individuals, respectively. While specificity could not be analyzed in this study, the in-house IgM ELISA captured all rRT-PCR-confirmed index cases, indicating excellent sensitivity. Furthermore, no DENV infections were detected by rRT-PCR in this study and the concurrent parent cohort study, suggesting that potential false-positive Zika IgM results due to cross-reactivity were unlikely. Finally, symptomatic Zika cases may have been erroneously categorized as asymptomatic, due to recall bias. However, as a whole, the questionnaires were likely to capture previous Zika-associated symptoms since: (i) of 23 cases that were ZIKV-positive at enrollment, 6 (rRT-PCR-negative IgM-positive) did report Zika-related symptoms; (ii) symptoms such as arthralgia may be persistent and re-occurring [32]; (iii) the participants were asked to report previously diagnosed ZIKV infections, likely capturing previous Zika-associated symptoms; and (iv) the 62% of asymptomatic infections are well within the previously reported range.

In conclusion, our ZIKV household study found that the majority of homes had ≥50% of Zika-positive members, demonstrating that ZIKV transmission was widespread among the enrolled households in urban Managua. Further, an S:A ratio of 14:23 (1:1.6) was estimated among the contacts, indicating that 62% of the infections were asymptomatic, thus contributing to silent transmission. Finally, the maximum number of days during which ZIKV RNA was detected was 7 days post-symptom onset in saliva and serum/plasma and 22 days in urine. Detecting ZIKV in saliva over a similar time period and concentration as serum/plasma indicates that saliva could potentially serve as a more accessible diagnostic sample. These findings provide information about detection of viral RNA, thus informing strategies for ZIKV detection in bodily fluids. The study also estimated urban ZIKV dissemination along with percent asymptomatic infections, assessing likelihood of silent transmission. Overall, the findings presented inform public health decision-making about Zika interventions, diagnostic methods and strategies, and vector control activities in Nicaragua, the region and globally.

## Supporting information

S1 Fig Directed acyclic graph (DAG) visualizing the causal assumptions for ZIKV infection risk factor analysis. Outcome was ZIKV infection and risk factors of interest were the following: sex, age, household size, water faucet location outside of the house, on-site water storage, recognizing mosquito larvae or pupae, fumigation, abatement use, trash collection services, water services and repellent use reports. SES; socioeconomic status. U; unknown.
(PDF)

S1 Table Zika virus (ZIKV)-positive index cases and contacts as determined by rRT-PCR in bodily fluids over study visits. (PDF)

S2 Table Total reported and enrolled household members among the study households (n = 33). (PDF)

S3 Table Duration of ZIKV RNA detection in bodily fluids of ZIKV rRT-PCR-positive study participants who presented Zika-associated symptoms. (PDF)

S4 Table Symptoms recorded for study participants and ZIKV status based on rRT-PCR and/or IgM laboratory results. (PDF)

S1 Checklist STROBE checklist. (PDF)
