# Supplementary figures and images for: Zika virus infection in Nicaraguan households
Source: PLoS Negl Trop Dis. 2018 May 31;12(5):e0006518. doi: 10.1371/journal.pntd.0006518 (PMC6014677; doi:10.1371/journal.pntd.0006518)

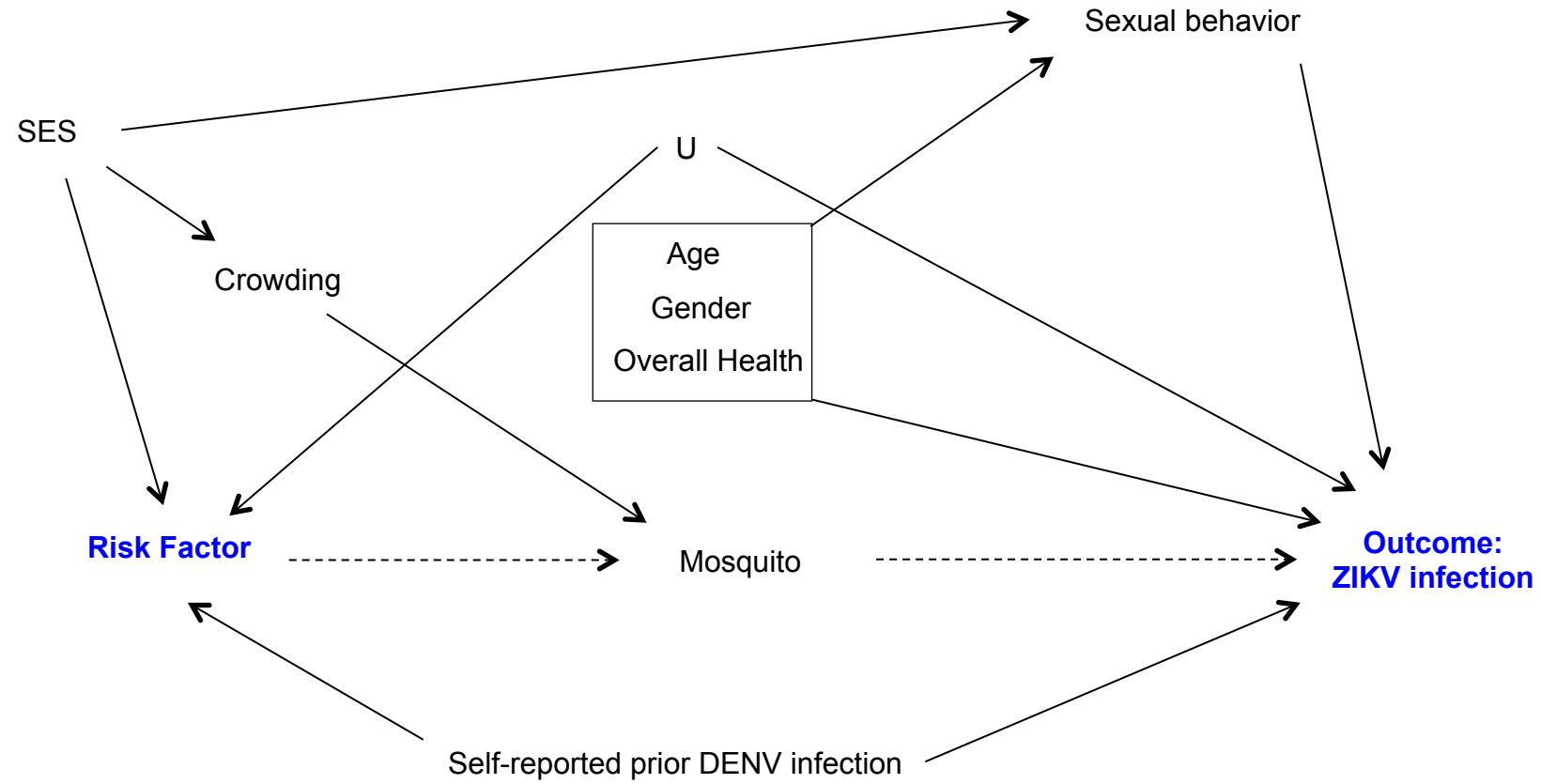

Supplement: S1 Fig — Outcome was ZIKV infection and risk factors of interest were the following: sex, age, household size, water faucet location outside of the house, on-site water storage, recognizing mosquito larvae or pupae, fumigation, abatement use, trash collection services, water services and repellent use reports. SES; socioeconomic status. U; unknown. (PDF) [file pntd.0006518.s001.pdf]
