# Supplementary material for: Zika virus infection in Nicaraguan households
Source: PLoS Negl Trop Dis. 2018 May 31;12(5):e0006518. doi: 10.1371/journal.pntd.0006518 (PMC6014677; doi:10.1371/journal.pntd.0006518)
Supplement: S1 Table — (PDF) [file pntd.0006518.s002.pdf]

Supplementary Table 1. Zika virus (ZIKV) positive index cases and contacts by rRT-PCR tested bodily fluids over study visits.

		Visit Number					
		**0**	**1**	**2**	**3**	**4**	**5**
ID	Fluid	Enrollment	Day 1	Day 3/4	Day 6/7	Day 9/10	Day 21
		2					24
150	Serum	5.9					N
		2	3	5	8	11	24
	Urine	N	3.7	5.7	8.9	N	N
		2	3	5	8	11	24
	Saliva	5.7	5	6.6	N	N	N
		3		6			
1105	Serum	5.3		N			
		3	6	9	12	14	27
	Urine	N	N	N	N	N	N
		3	6	9	12	14	27
	Saliva	5	5.5	N	N	N	N
		2		3			25
1619	Serum	N		3.8			N
		2	4	6	10	13	25
	Urine	5.2	5.3	5.9	4.4	N	N
		2	4	6	10	13	25
	Saliva	5.9	N	N	N	N	N
		1		4			24
4430	Serum	N		N			N
		1	4	6	9	11	24
	Urine	4.5	N	N	N	N	N
		1	4	6	9	11	24
	Saliva	4.1	N	N	N	N	N
		2		5			27
5091	Serum	N		N			N
		2	6	9	13		27
	Urine	5.2	6.6	3.3	N		N
		2	6	9	13	15	27
	Saliva	6.1	N	N	N	N	N
		2		5			26
5754	Serum	N		N			N
		2	5	8	10	13	26
	Urine	3.3	N	N	N	N	N
		2	5	8		13	26
	Saliva	4.9	N	N		N	N
		3		4			26
5805	Serum	N		N			N
		3	5	7	11	14	26
	Urine	3.4	4.7	N	N	4.2	N
		3	5	7	11	14	26
	Saliva	6.2	4.7	N	N	N	N
		2		6			27
5812	Serum	5.3		N			N
			6	9	11	15	27
	Urine		N	N	N	N	N
		3	6	9	11	15	27
	Saliva	4.7	N	N	N	N	N
		2		4			24
5831	Serum	7.2		4.3			N
		2	3	5	9	12	24
	Urine	N	4.9	4.5	N	4.2	N
		2	3	5	9	12	24
	Saliva	4.3	5.7	N	N	N	N
		3		7			30
5935	Serum	5.8		N			N
		5	9	12	15	17	30
	Urine	2.4	4.8	N	N	N	N
		5	9	12	15	17	30
	Saliva	5.7	N	N	N	N	N
		1		4			25

Days post-symptom onset

Vira loads (10log copies/ml)

N = Negative

Index case from cohort

Index case from surveillance

ZIKV pos. Contacts

Asympt. ZIKV pos. contact

P= Pregnant women

5942	Serum	N		N			N
		1	4	7	9	12	25
	Urine	3.1	3.8	N	N	N	N
		1	4	7	9	12	25
	Saliva	5.9	4.4	N	N	N	N
		2		5			26
6506	Serum	N		N			N
		3		8	11	15	26
	Urine	4.8		6.1	N	N	N
		3	5	8	11	15	26
	Saliva	5.3	6.2	N	N	N	N
		1		5			24
6697	Serum	6.5		N			N
		2	3	5	9	12	24
	Urine	N	5.7	5.4	4.8	3.8	N
		2	3	5	9	12	24
	Saliva	5.7	6.1	5.7	N	N	N
		2		4			26
6709	Serum	7.5		4.9			N
		2	4	6	10		26
	Urine	N	5.2	3.7	4.9		N
		2	4	6	10		26
	Saliva	5.1	6.3	6.5	N		N
		2		3			27
	Serum	N		N			N
7187		2	3	6	9	13	28
	Urine	3.6	4.7	N	N	N	N
		2	3	6	9	13	27
	Saliva	6.3	5.3	N	N	N	N
		2		3			25
7234	Serum	N		N			N
		2	4	7	10	13	25
	Urine	N	N	N	N	N	N
		2	4	7	10	13	25
	Saliva	5.7	N	N		N	N
		1		5			24
7253	Serum	6		N			N
		1	3	5	9	12	24
	Urine	N	5.2	5.2	4.5	N	N
		1	3		9	12	24
	Saliva	5.2	5.1		N	N	N
		2		4			24
7273	Serum	N		N			N
		2	3	6	9	12	24
	Urine	3.6	4.3	N	N	N	N
		2	3	6	9	12	24
	Saliva	5	5	4.1	N	N	N
		2		5			28
7341	Serum	5.4		N			N
		3	5	8	11	14	28
	Urine	4.3	5.2	5.3	5.3	5.3	N
		3	5	8	11	14	28
	Saliva	5.7	4.5	N	N	N	N
		1		6			27
7987	Serum	5		N			N
			6	9	11	15	27
	Urine		N	N	N	N	N
			6	9	11	15	27
	Saliva		N	N	N	N	N
		1		5			
8079	Serum	4.7		N			
		2	3			12	
	Urine	1.7	N			4	
			3				
	Saliva		4.4				
		1		5			29
8208	Serum	N		N			N

		1	5	8	11	13	29
	Urine	4.6	3.3	5.4	N	N	N
		1	5	8	11	13	29
	Saliva	5.6	N	N	N	N	N
		2		5			25
8280	Serum	5.5		N			N
		3	4	7	10	13	25
	Urine	N	3.2	4.2	4.2	4.7	N
		3	4	7	10	13	25
	Saliva	4.9	N	N	N	N	N
		1					23
8297	Serum	5.8					N
		1	2	4	7	10	23
	Urine	N	4.7	4.3	N	N	N
		1	2	4	7	10	23
	Saliva	5.4	6	5.4	4.7	N	N
		1		4			25
8594	Serum	N		2.9			N
		2	4	7	10	13	25
	Urine	4	4.5	4.4	N	N	N
		2	4	7	10	13	25
	Saliva	6.3	N	N	N	N	N
		2		4			26
9226	Serum	N		N			N
		2	5	8	11		26
	Urine	4.5	N	N	N		N
		2	5	8	11	13	26
	Saliva	6.9	5.2	N	N	N	N
		2		3			26
9419	Serum	N		N			N
		2	4	6	9	12	26
	Urine	3	5.2	N	N	N	N
		2	4	6	9	12	26
	Saliva	4.7	4.9	N	N	N	N
		2		4			25
9421	Serum	6.5		N			N
		2	4	7	10	13	25
	Urine	N	N	N	N	N	N
		2	4	7	10	13	25
	Saliva	3.2	4.4	N	N	N	N
		4					25
10605	Serum	P					N
P			4	7	10	13	25
	Urine		5.4	3.9	N	N	N
			4	7	10	13	25
	Saliva		N	N	N	N	N
		2					23
10606	Serum	P					N
P			2	5	8	11	23
	Urine		3.8	N	N	N	N
			2	5	8	11	23
	Saliva		N	N	N	N	N
		5					26
10731	Serum	P					N
P			5		11	14	26
	Urine		3.5		N	N	N
			5		11	14	26
	Saliva		N		N	N	N
		4					
11324	Serum	P					
P			4	7	10	13	

	Urine		4.8	4.7	N	N	
			4	7	10	13	25
	Saliva		N	N	N	N	N
		7					
11479	Serum	P					
			7	10	13	16	28
	Urine		5.4	5.3	3.9	N	N
			7	10	13	16	28
	Saliva		N	N	N	N	N
2339	Serum		N	N			N
	Urine		N	N	N	N	N
	Saliva		4.2	N	N	N	N
			**-1**	2			20
6226	Serum		6.7	N			N
			**-1**	2	5	7	20
	Urine		N	5.4	4.7	4.2	N
			**-1**	2	5	7	20
	Saliva		N	5.5	N	N	N
			1	4			22
8743	Serum		4.6	N			N
			1		7	10	22
	Urine		N		3.4	4.4	N
			1				
8912	Serum		8.1				
				3	7		22
	Urine			4.4	4.8		3.6
					7		22
	Saliva				N		N
			2	4			24
40009	Serum		N	N			N
			2	4	8	11	24
	Urine		5.5	6.1	4.9	4.1	N
			2	4	8	11	24
	Saliva		N	N	N	N	N
40012	Serum		N	N			N
	Urine		4.2	4.5	3.7	N	N
	Saliva		N	N	N	N	N
40041	Serum		4.7	N			N
	Urine		N	5.6	4.6	N	N
	Saliva		N	N	N	N	N
			2	5			
40042	Serum		N	N			
			2		8		23
	Urine		3.8		N		N
			2	5	8	12	23
	Saliva		5.2	N	N	N	N
			4	7			
40043	Serum		N	N			
			4	7	10	13	25
	Urine		5.2	4.7	N	N	N
			4	7	10	13	25
	Saliva		N	N	N	N	N
**40056**	Serum		N	N			N
	Urine		4.5	6.4			
	Saliva		N	N	N		N
			6	9			27
40061	Serum		N	N			N
			6	9	11	15	27
	Urine		5.8	4.8	N	N	N
			6	9	11	15	
	Saliva		N	N	N	N	
