# Supplementary material for: Zika virus infection in Nicaraguan households
Source: PLoS Negl Trop Dis. 2018 May 31;12(5):e0006518. doi: 10.1371/journal.pntd.0006518 (PMC6014677; doi:10.1371/journal.pntd.0006518)
Supplement: S2 Table — (PDF) [file pntd.0006518.s003.pdf]

Supplementary Table 2: Total reported and enrolled household members among the study households (n=33).

Household	Total reported household members	Total enrolled household members	Percent enrolled household members*
1	10	7	70
2	4	2	50
3	12	7	58
4	3	3	100
5	5	4	80
6	6	3	50
7	4	3	75
8	4	4	100
9	7	4	57
10	4	4	100
11	14	7	50
12	4	4	100
13	5	4	80
14	5	5	100
15	4	4**	75
16	7	4	57
17	5	3	60
18	6	5	83
19	12	7	58
20	16	5	31
21	6	3	50
22	5	3	60
23	4	2	50
24	5	3	60
25	6	5	83
26	4	3	75
27	5	2	40
28	6	6	100
29	6	4	67
30	13	8	62
31	19	5	26
32	6	5	83
33	5	4	80
**Total**	**227**	**142**	**63**

*Household members who were not enrolled were not present (at home) during enrollment visit.
**One enrolled contact did not answer the individual questionnaire but fulfilled the other study requirements, such as symptom questionnaires and sample donation.
