# Supplementary material for: Zika virus infection in Nicaraguan households
Source: PLoS Negl Trop Dis. 2018 May 31;12(5):e0006518. doi: 10.1371/journal.pntd.0006518 (PMC6014677; doi:10.1371/journal.pntd.0006518)
Supplement: S3 Table — (PDF) [file pntd.0006518.s004.pdf]

Supplementary Table 3. Duration of ZIKV RNA detection in bodily fluids of ZIKV rRT-PCR-positive study participants who presented Zika-associated symptoms.

	**Serum/ Plasma**	**Saliva**	**Urine**
N[1]	25	51	86
Range[2]	6	6	21
Mean[3]	2.56	3.10	5.94
Standard Deviation	1.53	1.50	3.80
Median[4]	2.00	3.00	5.00
Variance	2.34	2.25	14.55

[1]Number of ZIKV rRT-PCR-positive samples from Zika cases that were included in the analysis of duration of ZIKV RNA detection
[2]Range of duration of ZIKV positivity in days post-symptom onset
[3]Mean duration of ZIKV positivity in days post-symptom onset
[4]Median duration of ZIKV positivity in days post-symptom onset
