# Supplementary material for: Zika virus infection in Nicaraguan households
Source: PLoS Negl Trop Dis. 2018 May 31;12(5):e0006518. doi: 10.1371/journal.pntd.0006518 (PMC6014677; doi:10.1371/journal.pntd.0006518)
Supplement: S4 Table — (PDF) [file pntd.0006518.s005.pdf]

**Supplementary Table 4.** Symptoms recorded for study participants and ZIKV status based on rRT-PCR and/or IgM laboratory results.

| | Subject ID | ZIKV Positive | Symptom 1 | Symptom 2 | Symptom 3 | Symptom 4 | Symptom 5 | Symptom 6 |
|---|---|---|---|---|---|---|---|---|
| 1 | 5405 | No | Cough | | | | | |
| 2 | 6154 | No | Cough | Nausea | Vomit | Continous abdominal pain | | |
| 3 | 6226 | Yes | Rash | | | | | |
| 4 | 6454 | No | Cough | Rhinorrhea | | | | |
| 5 | 6941 | No | Fever | Cough | Rhinorrhea | Headache | Throatpain | |
| 6 | 7155 | Yes | Arthralgia | | | | | |
| 7 | 7508 | No | Cough | | | | | |
| 8 | 7654 | No | Cough | Rhinorrhea | | | | |
| 9 | 8743 | Yes | Fever | Rash | | | | |
| 10 | 8912 | Yes | Fever | Rash | | | | |
| 11 | 40004 | Yes | Nausea | Headache | Ophthalmoplegia | | | |
| 12 | 40009 | Yes | Fever | Arthralgia | Rash | Rhinorrhea | | |
| 13 | 40011 | No | Cough | Rhinorrhea | Throatpain | | | |
| 14 | 40014 | No | Fever | Headache | Continous abdominal pain | Diarrhea | | |
| 15 | 40015 | No | Headache | Vomit | Diarrhea | Arthralgia | Retroocular pain | |
| 16 | 40024 | Yes | Pruritus | Arthralgia | | | | |
| 17 | 40027 | No | Cough | Throatpain | Fever | Headache | | |
| 18 | 40030 | No | Cough | Rhinorrhea | Throatpain | | | |
| 19 | 40038 | Yes | Arthralgia | | | | | |
| 20 | 40039 | No | Headache | | | | | |
| 21 | 40042 | Yes | Fever | Headache | Rash | Difficulty breathing | Arthralgia | Cough |
| 22 | 40043 | Yes | Headache | Rash | Fever | Cough | | |
| 23 | 40052 | Yes | Rash | Shivers | Arthralgia | | | |
| 24 | 40060 | No | Pruritus | | | | | |
| 25 | 40061 | Yes | Rash | | | | | |
| 26 | 40064 | No | Arthralgia | Myalgia | Cefalea | Poor general health | | |
| 27 | 40066 | Yes | Fever | | | | | |
| 28 | 40067 | No | Fever | Poor general health | Arthralgia | | | |
| 29 | 40071 | No | Rash | Pruritus | | | | |
| 30 | 40072 | No | Arthralgia | | | | | |
| 31 | 40074 | No | Fever | | | | | |
| 32 | 40075 | No | Fever | Headache | Poor general health | | | |
| 33 | 40077 | Yes | Headache | | | | | |
| 34 | 40080 | No | Headache | | | | | |
